# What are the reasons for refusing a COVID-19 vaccine? A qualitative analysis of social media in Germany

**DOI:** 10.1186/s12889-022-13265-y

**Published:** 2022-04-27

**Authors:** Jana Fieselmann, Kübra Annac, Fabian Erdsiek, Yüce Yilmaz-Aslan, Patrick Brzoska

**Affiliations:** 1 Faculty of Health, School of Medicine, Health Services Research, Witten/Herdecke University, Witten, Germany; 2 Faculty of Health Sciences, Nursing and Health Services Research, Bielefeld University, Bielefeld, Germany; 3 Faculty of Health Sciences, Epidemiology & International Public Health, Bielefeld University, Bielefeld, Germany

**Keywords:** SARS-CoV-2, COVID-19, Vaccination, Trust in vaccine, Compliance, Pandemic, Social media analysis, Germany

## Abstract

**Background:**

Vaccination against COVID-19 has been available in Germany since December 2020. However, about 30% of the population report not wanting to be vaccinated. In order to increase the willingness of the population to get vaccinated, data on the acceptance of vaccination and its influencing factors are necessary. Little is known about why individuals refuse the COVID-19 vaccination. The aim of this study was to investigate the reasons leading to rejecting vaccination, based on posts from three social media sites.

**Methods:**

The German-language versions of Instagram, Twitter and YouTube were searched regarding negative attitudes towards COVID-19 vaccination. Data was extracted until a saturation effect could be observed. The data included posts created from January 20, 2020 to May 2, 2021. This time frame roughly covers the period from the first reports of the spread of SARS-CoV-2 up to the general availability of vaccines against COVID-19 in Germany. We used an interpretive thematic approach to analyze the data and to inductively generate codes, subcategories and categories.

**Results:**

Based on 333 posts written by 323 contributing users, we identified six main categories of reasons for refusing a COVID-19 vaccination: Low perceived benefit of vaccination, low perceived risk of contracting COVID-19, health concerns, lack of information, systemic mistrust and spiritual or religious reasons. The analysis reveals a lack of information among users and the spread of misinformation with regard to COVID-19 and vaccination. Users feel inadequately informed about vaccination or do not understand the information available. These information gaps may be related to information not being sufficiently sensitive to the needs of the target group. In addition to limited information for the general population, misinformation on the internet can also be an important reason for refusing vaccination.

**Conclusions:**

The study emphasizes the relevance of providing trustworthy and quality-assured information on COVID-19 and COVID-19 vaccination to all population groups. In addition, vaccinations should be easily accessible in order to promote the population’s willingness to be vaccinated.

## Background

In addition to preventive measures such as social distancing, mandatory face coverings in public settings and intensified hygiene measures, vaccination considerably reduces the risk of contracting COVID-19 [1]. Vaccines against COVID-19 have been available in Germany since December 2020. Since May 2020, the COSMO COVID-19 Snapshot Monitoring project has been investigating the willingness of the population in Germany to receive COVID-19 vaccination. Their surveys show that the proportion of those willing to be vaccinated has steadily declined since the beginning of the survey, falling from 79% in mid-April 2020 to 48% in mid-December 2020 [2, 3]. It again increased to 57% at the end of December 2020 [2, 3]. At the beginning of March 2021, the vaccination readiness of the respondents was 68% [4]. As of mid-December 2021, 70.9% of the population in Germany are fully vaccinated [5]. 61% of those still unvaccinated report that they do not want to get vaccinated and 26% remain hesitant. Only 13% of those vaccinated plan to receive a vaccination shot in the near future [5].

In order to increase the willingness of the population to get vaccinated, data on the acceptance of vaccination and its influencing factors are necessary [6]. Findings on vaccine acceptance in the context of other infectious diseases, such as influenza or measles, are only partially applicable to COVID-19 due to the novelty of the disease, its long-term effects, its consequences on social life, and the widespread use of mRNA vaccines as a new type of vaccines.

According to the Health Belief Model, preventive behavior such as receiving a vaccination depends on the perceived risks of disease as well as the perceived effectiveness, benefits and costs associated with this behavior [7]. The COSMO COVID-19 Snapshot Monitoring shows that confidence in the safety of the vaccines used is the most relevant factor for a positive attitude of the population towards vaccination. In addition, the intention to get vaccinated is higher among older individuals, individuals whose families generally support vaccination, and individuals who are worried about infection [8]. A German telephone survey on the willingness to receive a COVID-19 vaccination also revealed that belonging to a population group at high risk for a severe course of COVID-19, a previous COVID-19 diagnosis and the expectation of severe complications are important factors associated with positive attitudes towards a COVID-19 vaccination [9]. Concerns about potential side effects were identified as one of the reasons for refusing vaccination [9]. According to a study by Taylor et al., perceived low benefits, concerns about potential long-term consequences, criticism towards financial interests of pharmaceutical companies and a preference for natural immunity against COVID-19 were also associated with low willingness to get vaccinated [10].

In the present study, available findings on COVID-19 vaccine hesitancy are complemented by insights from the perspective of social media users in Germany. This contributes to existing knowledge by exploring attitudes of a population group that is often difficult to reach through other channels. Insights gained through an analysis of social media posts can therefore significantly inform public health measures aiming to promote COVID-19 vaccination in this population group.

## Methods

The German-language versions of the online platforms Instagram, Twitter and the comments section of YouTube were searched for reported reasons for rejecting a COVID-19 vaccination. The search terms used to identify relevant postings were the following keywords in German: "Corona vaccination", "COVID-19 vaccination", "SARS-CoV-2 vaccination", "BioNTech-Pfizer", "Moderna", "AstraZeneca", "Johnson & Johnson". The search terms were not combined using operators. The keywords were entered in the search field of the respective platforms one by one. Relevant posts in German language were extracted into a text document. Posts in closed groups were not included. Data was extracted until a saturation effect could be observed. Inclusion criteria aside from the aforementioned keywords were: the posts had to be written in German language and had to report hesitancy or refusal with respect to the COVID-19 vaccination. The data included posts created from January 20, 2020 to May 2, 2021. This time frame roughly covers the period from the first reports of the spread of SARS-CoV-2 up to the general availability of vaccines against COVID-19 in Germany. This period includes the initial stages of the development of the respective vaccines, their approval and introduction in Germany through specialized vaccination centers and the subsequent extension of the vaccination strategy to also include vaccination through general practitioners and family physicians. The beginning of May 2021 also marked the end of the prioritization of specific vulnerable groups for receiving the Vaxzevria® vaccine, the first widely available vaccine against COVID-19 in Germany [11]. Facebook, Instagram and Twitter are the most widely used freely accessible social media forums in Germany, and accordingly the highest numbers of posts are available on these sites, therefore these social media forums were selected [12, 13]. Moreover, social media are frequently used communication channels to disseminate both high-quality information and misinformation [14].

A total of 333 posts written by 323 contributing users were used in our analysis, including 121 posts from Instagram, 84 from Twitter and 128 from YouTube (Table 1). We used an interpretive thematic approach to analyze the data and to inductively generate codes, subcategories and categories [15]. Relevant text sections were marked in the text document and then coded. The data was initially coded by the main author and discussed and consented with the other authors to minimize the risk of researcher bias. Sections with similar coding were grouped into main categories, which were divided into subcategories based on their content. Finally, codes were summarized and are presented in an explanatory manner. To obtain codes and derive categories and subcategories, we used the MAXQDA software.

**Table 1:** Reasons for refusing COVID-19 vaccination. Identified categories based on 333 posts in social media (Instagram, Twitter, YouTube) and number of posts identified in each case

Category	Number of postings
Low perceived benefits	30
Low subjective risk	48
Health concerns	89
Information deficits	87
Systemic mistrust	76
Spiritual or religious beliefs	3

For our analysis, freely accessible information from posts on social media platforms was used, which was considered open data in comparable previous studies [16]. In order to preserve the anonymity of the social media users, user names were replaced with numbered identifiers (e.g., User 01). The language of the quotations was also adapted to prevent identification through search engines. Incorrect spelling was corrected and abbreviations were spelled out, while ensuring to preserve the meaning of the post.

## Results

Our analysis revealed six main categories of reasons for refusing a COVID-19 vaccination. Reasons include low perceived benefits of getting vaccinated, a low subjective risk, concerns of potential adverse effects from the vaccine, poor health literacy, mistrust and spiritual and religious beliefs (see Table 1).

### Low perceived benefits

Several posts show that social media users did not trust the newly developed mRNA-based vaccines or had some reservations about gene-based vaccines in general. According to these posts, users thought that vaccination with mRNA-vaccines had not yet been sufficiently investigated or that they were not as effective as attenuated or inactivated vaccines, which is why they did not consider vaccination with the existing vaccines to be necessary or sensible.

"Pfizer's vaccines and other vaccines against COVID-19 are experiments, not vaccines. These are novel genetic technologies that have never been used on humans before. An mRNA molecule can never stimulate the immune system the way a vaccination can." [User 33]

### Low subjective risk

Furthermore, the analysis shows that users viewed their personal risk of getting infected with COVID-19, suffering from a severe course of the disease and developing serious complications from an infection as low. Therefore, vaccination was not regarded necessary. Mild symptoms, a young age and a good subjective health status were reported as relevant factors leading to that evaluation.

"I already had Corona, I only had a slight cough for two days, it didn't bother me at all. I'd rather get Corona than have anything injected into my blood. Everyone as they like." [User 140]

In addition, some users emphasized that their own immune system was strong enough to deal with a possible infection and therefore they did not need vaccination. According to their own statements, some of the users relied on preventive and supportive measures like a balanced diet or taking supplemental vitamins to bolster up their immune system, rendering vaccination, in their opinion, unnecessary.

"I am asthmatic, but I would never be vaccinated against Corona. I don't have to weigh that. We have an immune system. I live a healthy life with lots of vitamins. I don't deny Corona, but I'm not afraid of it." [User 47]

Moreover, some users on social media stated that prior infections with COVID-19 made them immune to reinfection, including immunity to mutations of the virus, and therefore a vaccination was not necessary. In line with that, some users believed that a prior infection offered more natural protection than the vaccines.

"No one needs this vaccination, because once you have Corona you are immune." [User 4]

### Health concerns

Another reason to refuse vaccination were users' concerns about various potential side effects and possible vaccine-related damage. Some users justified rejecting vaccination citing the lack of long-term studies and insufficient reliable information about side effects and consequential damages. Among others, these fears were related to the risk of getting cancer, changes and damages to their genetic makeup, infertility and death. These concerns were often associated with past vaccine and drug scandals.

"I don't get vaccinated, I'm afraid of side effects. Thalidomide, for example, should not be ignored either. There are no long-term studies, but everyone should do what they want." [User 121]

Users emphasized their objection especially regarding the possible approval of vaccine use for women during pregnancy. This was based on the lack of data supporting safe use in these cases and concerns about the effects of vaccination on the unborn child.

"Who does that?! I'm sorry, that's irresponsible. No one knows what happens to the unborn child, no one can take that responsibility on themselves! It's a pure human experiment." [User 296]

Our analysis also showed that vaccination was refused due to other pre-existing health conditions and allergies, as only little information was available on possible interactions between existing health impairments and COVID-19 vaccines. Personal experiences with physical reactions or vaccine damages from past vaccinations were further reported reasons that led to rejection of the vaccines.

"I am chronically ill and take a lot of medication, I have very great respect for vaccination. There is no data on the side effects and interactions in connection with the medication. That is too meagre for me." [User 160]

### Information deficits

Another reason for users refusing vaccination was that some did not feel sufficiently informed about the vaccination and that the available information was perceived as incomprehensible.

“No, I don't feel informed enough because the text is too difficult to understand." [User 124]

This lack of transparent and user-oriented information in some cases resulted in the spread of misinformation and conspiracy theories. The lack of knowledge led to a general mistrust and a negative attitude towards information on the disease itself and vaccines among some of the users. These beliefs, which were mostly based on misinformation or conspiracy theories, led to strong downplaying or denial of COVID-19 among users and a subsequent lack of willingness to get vaccinated.

"Corona vaccination is seen as protection. However, these vaccinations have the opposite effect. They are killings with the intention of reducing the world's population. Survive or die together? Which would also be romantic." [User 261]

### Systemic mistrust

Mistrust in authorities, political stakeholders or in representatives of the pharmaceutical industry also played an important role. There were doubts about the reliability and integrity of information and the intentions of certain groups, organizations or institutions in promoting vaccination, which users attributed to previous misconduct. For example, users were convinced that the pharmaceutical industry had a mere financial interest in promoting vaccination against COVID-19.

"Unfortunately, I cannot trust the pharmaceutical industry, as much as I would like to. I would get vaccinated, but my mistrust is far too great. I have also not yet received anything that would build my trust. In the past, the pharmaceutical industry has acted unethically and immorally and knowingly harmed people. They have put their sales first, for example with the Duogynon scandal. [...]" [User 140]

Furthermore, the rapid development and approval of the vaccines compared to previous vaccines against other diseases was another reason given by users for refusing vaccination. They expressed concern that the vaccines were not sufficiently tested and that long-term negative physical consequences could not be ruled out. The partial emergency approval of the vaccines also led to concerns.

"I'm not going to have it injected. Normally a vaccine is researched for years. And now I'm supposed to get injected with something that was mixed together in a very short time?" [User 186]

In addition, vaccines from specific manufacturers were sometimes rejected. Users justified this with differences in perceived effectiveness and suspected side effects of vaccines from certain manufacturers. The respective country of development or production also played a role in rejecting these vaccines.

"I'd rather die before I get vaccinated with the Russian or Chinese vaccine." [User 70].

### Spiritual or religious beliefs

Spiritual or religious beliefs, such as the protection by God or the protective effect of precious stones, also led to a refusal of vaccination against COVID-19 by some users.

"I don't believe in this vaccination and this vaccination will not help us, I believe in God, in Jesus Christ and only he can help us, save us and protect us from this virus." [User 219]

## Discussion

The COVID-19 pandemic has had far-reaching consequences for the society at large, with people having to reduce social contacts or even spending prolonged periods of time in isolation and quarantine as a result of infection control measures. This may have had a negative impact on the mental health of those affected, leading to an increase in depression and anxiety [17, 18]. Vaccination is one of the most important strategies for the long-term management of the COVID-19 pandemic [19]. Given that considerable proportions of the populations in many countries are still hesitant to get vaccinated [20], insights into reasons for poor vaccine acceptance are needed in order to inform public health measures aiming to further increase COVID-19 vaccination rates in the population.

The present study investigated reasons that users of Instagram, Twitter and YouTube presented for rejecting vaccination against COVID-19. In our analysis, six main categories of reasons were identified, which reflect different opinions and views.

Our analysis points to a lack of information among users and the spread of misinformation with regard to COVID-19 and vaccination. Users feel inadequately informed about vaccination or do not understand the information available. These information gaps may be related to information not being sufficiently sensitive to the needs of the target group. For example, information provided by the Robert Koch-Institute in the Frequently Asked Questions section on the topic of COVID-19 and vaccination (as of 28/12/2021) is not barrier-free and is available neither in simple language nor in major foreign languages spoken by immigrants in Germany [21]. Information from the Federal Ministry of Health regarding questions and answers on COVID-19 vaccination is available in sign language, simple language and English (as of 28/12/2021). However, information materials in other languages frequently spoken in Germany, such as Turkish or Russian, are missing [22]. The Federal Ministry of Health also refers to the Paul Ehrlich Institute for information on potential side effects of COVID-19 vaccination, which are not easily comprehensible by laypersons [23]. This demonstrates the need for more low-threshold and target group-specific communication addressing laypersons as well as people who are not fluent in German. One approach to address these aspects is a comprehensive booklet on COVID-19 vaccination which was jointly published only recently by the Federal Centre for Health Education and the Robert Koch-Institute and which is available in several languages (https://www.bzga.de/infomaterialien/impfungen-und-persoenlicher-infektions-schutz/3537/das-impfbuch-fuer-alle-1/). In addition to limited information for the general population, misinformation on the internet can also be an important reason for refusing vaccination. Betsch et al. confirm our findings by noting that even a short visit to a website with vaccine-skeptical content can influence the perception of vaccination risks and the willingness to get vaccinated. Consequently, perceived health risks from vaccination may increase and the intention to get vaccinated as well as perceived risks in case of non-vaccination may be reduced [24]. While the German government has recently introduced laws to address hate speech and misinformation in social media, the effectiveness and impact of these laws have yet to be evaluated. These findings emphasize the relevance of low-threshold, accessible, transparent and quality-controlled information on COVID-19 and vaccination. A low perceived benefit of the vaccination and a low subjective risk of getting COVID-19 can also be counteracted by providing laypersons with easily understandable and transparent information about the etiology as well as the course of the disease and possible sequelae of an infection such as Long-COVID.

Moreover, users may refuse a COVID-19 vaccination due to systemic mistrust in authorities, political stakeholders or representatives of the pharmaceutical industry. A politically and economically independent information center could contribute to counteracting system-relevant mistrust and strengthen trust in the information communicated. This in turn could help increase vaccine acceptance. For a more general, long-term perspective, efforts to increase political participation, more transparency concerning the approval of pharmaceutical products, and targeted measures to increase health literacy could aid in improving trust in political and pharmaceutical actors and reduce the effect of targeted misinformation.

Our findings are in line with studies from other countries, which have shown that, amongst others, poor perception of government and public health responses to the pandemic, concerns about vaccine side effects and safety, and unfavorable illness perceptions about COVID-19 such as a low perceived risk of infection are relevant reasons for poor uptake of COVID-19 vaccines [25–28]. With regard to vaccinations related to other diseases, similar reasons as in our and previous research on COVID-19 vaccine hesitancy were reported in other studies, such as a lack of confidence in vaccinations and a low perceived risk of contracting the disease [29–32]. Still, because of the novelty of COVID-19, its tremendous impact on societies all over the world, the widespread use of mRNA vaccines as a new type of vaccines and discussions about mandatory vaccination as a strategy for the long-term management of the pandemic, further insights into individuals’ perception about COVID-19 vaccines are warranted.

The aforementioned strategies aiming to promote COVID-19 vaccination uptake need to be further strengthened and evaluated with regard to their effectiveness. In addition, the dissemination of information should be extended to multiple settings using different types of media [33]. Furthermore, involving trusted individuals in the communication of information can help to address misinformation [34].

To the best of our knowledge, this is the first study in Germany to investigate the readiness to get vaccinated against COVID-19 from the perspective of social media users. The current discussion around vaccination against COVID-19 in Germany differs from prior issues of vaccine hesitancy and skepticism towards other vaccines in several respects: (I) the spread of a newly emerging disease such as COVID-19 and its impact around the globe are generally seen as unprecedented in modern times and have overwhelmed health systems in several countries, (II) the resulting pressure to find ways to stop the spread of SARS-CoV-2 and to combat the pandemic has led to accelerated development and approval of respective vaccines, (III) the novel use of mRNA-based vaccines.

Our study has several limitations that need to be considered. While the number of users of social media is growing, potential selection effects in the user base of the platforms used could mean that our results cannot be representative for the overall population in Germany [35]. Accordingly, it is unclear to what extent our results are applicable to other population groups that do not use social media. Instead, our study should be understood as an extension of previous findings by including the specific perspective of social media users. In addition, no socio-demographic data such as age or sex could be included in the analysis, as these were not available. We also restricted our search strategy to keywords identified in the posts. Future research could also include hashtags and other types of identifiers to increase the comprehensiveness of the extracted information [36]. Furthermore, it should be taken into account that our focus was only placed on three frequently used social networks. We also could not examine changes in attitudes and perceptions over time due to the high number of users with only single posts related to vaccine hesitancy. Finally, when interpreting and comparing our findings to other studies, it is important to keep in mind that only posts from the period January 20, 2020 to May 2, 2021 were included in our analysis. COVID-19 vaccination has been available in Germany since December 2020. In this context, it would be worthwhile for future research to investigate whether and to what extent vaccination attitudes and the willingness to get vaccinated have developed over time and which factors affect that development.

## Conclusion

Following the Health Belief Model, individuals use preventive behavior when they feel at risk from a health threat, consider the preventive behavior as effective, or think the benefits of the behavior outweigh the cost (including health risks associated with the preventive behavior itself) [7]. Accordingly, vaccination services should be offered at the lowest perceived cost possible, so that the personal cost–benefit assessment is in favor of COVID-19 vaccination. Consequently, vaccination must be easily accessible and without much bureaucratic effort in order to promote the willingness to be vaccinated. For example, the 47th wave of the COSMO COVID-19 Snapshot Monitoring recommends a focus on vaccination in the workplace and in the educational sector [37]. In addition, target group-specific education about the disease and its long-term consequences could promote vaccination readiness and acceptance. Education about the importance and benefits of vaccination, such as protection against severe courses of the disease, protection of others and gradual return to normality, can also have a positive impact on vaccination attitudes [37]. Further studies of diverse groups of people and minorities in Germany are recommended in order to also reach those who avoid the topic of COVID-19 and the corresponding vaccination. The findings regarding the reasons for rejection can be used as a basis for future decisions in the context of the COVID-19 pandemic, as well as for possible future crises to address the build-up of mistrust and the generation of misinformation with appropriate counter measures. In this respect, also the information on vaccination should generally be made available in several languages to address the spread of misinformation.

## Data Availability

The data used analyzed during the study are not publicly available due to privacy and data protection concerns (potential de-anonymization) but are available from the corresponding author upon reasonable request.
